# An Efficient Pairing-Free Ciphertext-Policy Attribute-Based Encryption Scheme for Internet of Things

**DOI:** 10.3390/s24216843

**Published:** 2024-10-24

**Authors:** Chong Guo, Bei Gong, Muhammad Waqas, Hisham Alasmary, Shanshan Tu, Sheng Chen

**Affiliations:** 1 College of Computer Science, Beijing University of Technology, Beijing 100124, China; chongguo@emails.bjut.edu.cn (C.G.); gongbei@bjut.edu.cn (B.G.); sstu@bjut.edu.cn (S.T.); 2 School of Computing and Mathematical Sciences, Faculty of Engineering and Science, University of Greenwich, London SE10 9LS, UK; 3 School of Engineering, Edith Cowan University, Joondalup, WA 6027, Australia; 4 Department of Computer Science, King Khalid University, Abha 62529, Saudi Arabia; alasmary@kku.edu.sa; 5 School of Electronics and Computer Science, University of Southampton, Southampton SO17 1BJ, UK; sqc@ecs.soton.ac.uk

**Keywords:** ciphertext-policy attribute-based encryption, pairing-free, access control, Internet of Things

## Abstract

The Internet of Things (IoT) is a heterogeneous network composed of numerous dynamically connected devices. While it brings convenience, the IoT also faces serious challenges in data security. Ciphertext-policy attribute-based encryption (CP-ABE) is a promising cryptography method that supports fine-grained access control, offering a solution to the IoT’s security issues. However, existing CP-ABE schemes are inefficient and unsuitable for IoT devices with limited computing resources. To address this problem, this paper proposes an efficient pairing-free CP-ABE scheme for the IoT. The scheme is based on lightweight elliptic curve scalar multiplication and supports multi-authority and verifiable outsourced decryption. The proposed scheme satisfies indistinguishability against chosen-plaintext attacks (CPA) under the elliptic curve decisional Diffie–Hellman (ECDDH) problem. Performance analysis shows that our proposed scheme is more efficient and better suited to the IoT environment compared to existing schemes.

## 1. Introduction

The development of the Internet of Things (IoT) and the advancement of 5G technology have greatly reduced the deployment costs of IoT devices and facilitated the application and expansion of the IoT [1,2]. As the number of IoT devices continues to grow and application scenarios become more complex and diverse, the volume of data generated in the IoT has surged. However, most current IoT devices are equipped with low-voltage and low-power processors, small storage space, and relatively low energy storage. This makes efficient data sharing and low-energy-consumption data processing critical in IoT [3,4]. Reducing the computational costs of a device is key to lowering its energy consumption, improving operational stability, and extending its lifecycle [5,6].

As an important innovation in the development of the Internet, cloud computing technology is currently very mature and widely used. Cloud servers provide abundant computing resources and massive storage space, allowing IoT devices to offload complex computational tasks to the cloud and store data for extended periods [7]. With the help of cloud servers, data can be conveniently shared between devices [8,9]. Utilizing cloud computing to perform specific computing tasks in the IoT can improve the speed of data processing, reduce computational costs, and extend the lifecycle of devices [10]. However, cloud service providers (CSPs) are untrusted third-party organizations, and the cloud computing services and cloud storage services they provide are uncontrollable for data owners [11,12,13,14]. To eliminate the security risks associated with leakage, data should be encrypted before being uploaded to hide sensitive information [15]. Data sharing in the IoT involves a many-to-many relationship. From a data perspective, sharing one piece of data is a one-to-many relationship. However, traditional cryptographic schemes, such as advanced encryption standard (AES) and Rivest–Shamir–Adleman (RSA) are only suitable for one-to-one ciphertext sharing, requiring data to be encrypted multiple times for different recipients. This results in an increased number of encryption tasks, leading to higher computational costs and energy consumption for IoT devices. Furthermore, storing these multiple ciphertexts in the cloud can significantly increase the required storage space.

Attribute-based encryption (ABE) schemes can effectively solve the above problem [16,17,18]. They can set up an attribute-based access structure by defining a set of attributes which can describe the identity of legitimate users under the premise that the data visitors are unknown [19,20,21]. The user can successfully decrypt the ciphertext only when the attributes satisfy the access structure. Ciphertext-policy ABE (CP-ABE) was proposed by Bethencourt et al. [22] based on the ABE of [23]. CP-ABE fuzzes users with attributes and achieves fine-grained access control with the access structure, making it suitable for one-to-many data sharing relationships between huge numbers of devices in the IoT [24]. In CP-ABE, the ciphertext is associated with an access policy and the key is generated based on a set of attributes. This allows the data owner to establish the access policy autonomously and share the data with various users while safeguarding the access control of the data [16,25]. Currently, most CP-ABE schemes use bilinear pairing as the primary operation [26]. However, bilinear pairing is a complex operation that is considered to have the highest computational cost in pairing-based cryptographic protocols [27]. Therefore, CP-ABE schemes based on bilinear pairing suffer from long encryption times and high-energy consumption. These limitations render their direct application a challenging task in IoT environments, where computational resources are scarce and energy storage is limited.

To address these challenges, the scheme [28] keeps the length of the ciphertext constant and independent of the number of attributes chosen, thus reducing the need for bilinear pairings during decryption. In addition, outsourcing complex decryption computations to cloud servers can further reduce the computational burden on devices [27,29]. In recent years, several elliptic curve cryptography (ECC)-based ABE schemes without pairing have been proposed [30,31,32,33,34,35]. On the same elliptic curve, the computational cost of a single bilinear pairing is more than twice that of the scalar multiplication method, while the computational cost of an asymmetric bilinear pairing is 10–40 times that of the scalar multiplication method. Replacing bilinear pairing with scalar multiplication as the main operation of CP-ABE can dramatically reduce the overall computational cost of the system, which provides a novel and feasible approach for the application of CP-ABE in IoT. Against this background, this paper proposes an efficient data-sharing scheme based on pairing-free CP-ABE for IoT. The main contributions of this work are summarized as follows.

(1) We propose a pairing-free CP-ABE scheme for the IoT that uses elliptic curve scalar multiplication as the primary operation. This design retains the fine-grained access control features of CP-ABE while significantly reducing computational complexity, making it more suitable for IoT devices with limited computing resources.
(2) Our scheme establishes multiple attribute authorities (AAs) to decentralize the attribute management. In this way, our scheme can avoid the system bottleneck and key escrow problems caused by a single AA in traditional CP-ABE schemes, and guarantee rapid response to requests from a massive amount of IoT devices.
(3) We ensure data security with the hybrid cryptographic method, encrypting data with symmetric cryptographic algorithms and encrypting keys with CP-ABE. Moreover, linear secret sharing scheme (LSSS) is adopted to enhance the expression of the access policy and to provide flexible access control.
(4) Our scheme supports verifiable outsourced decryption. With outsourced computing, only a small amount of computation is required to decrypt the ciphertext on the IoT device, which effectively reduces the decryption cost. Before decrypting with the symmetric key, the device can determine whether the data has been tampered through the integrity verification function.
(5) We conducted a detailed security analysis and performance analysis of the proposed scheme and the results prove that it is both secure and efficient.

The remainder of this paper is organized as follows. Section 2 introduces the related work on ABE, and some preliminaries are given in Section 3. Section 4 describes the system model and the security model of our scheme, while an efficient pairing-free CP-ABE scheme for the IoT is proposed in Section 5. Section 6 and Section 7 are devoted to the security analysis and performance analysis of our scheme, respectively. Our conclusions are presented in Section 8.

## 2. Related Work

### 2.1. Typical ABE Schemes

Amit and Waters [23] extended ABE by abstracting the features of a user’s identity based on the identity-based encryption (IBE) [36]. By defining specific identities through a set of attributes, ABE overcomes the limitations of IBE that use a single identification information to determine users’ identities, which enables fine-grained access control for users while still providing privacy protection for them. Although the work [23] successfully introduced the concept of attributes, access control was implemented by gate access structure, which suffers from inefficiency as well as limitations. Goyal et al. [37] first proposed key-policy ABE (KP-ABE). KP-ABE embeds an access policy in the key, and correlates the set of attributes with the ciphertext, so that the ciphertext can be decrypted only if the set of attributes satisfies the access policy. To make the expression of the access structure more flexible, the work [37] used a monotonic access tree.

Bethencourt et al. [22] proposed CP-ABE with the opposite structure to KP-ABE. Compared to KP-ABE, CP-ABE matches ciphertexts with keys and better reflects the concept of abstracting users into roles. This makes it easier to provide flexible and fine-grained control over user access to data. However, CP-ABE of [22] also uses a monotonic access tree. Ibraimi et al. [38] proposed a CP-ABE scheme that supports access structures represented by Boolean operator formulas. Although this scheme has low computational efficiency, it successfully removes the restriction where only the access tree can be used. Waters [39] proposed an LSSS and designed a CP-ABE scheme with LSSS. Compared to the previous schemes, the scheme of [39] provides more fine-grained access control to users, but only proves the security under the decisional parallel bilinear Diffie–Hellman exponent assumption.

Nishide et al. [40] first implemented hiding the access policy, making it impossible for any user to obtain any information about the access policy associated with the encrypted data from the ciphertext, and proved the security of the scheme under the decisional bilinear Diffie–Hellman (DBDH) and discrete logarithm (DL) assumptions. Lewko and Waters [19] proposed a multi-authority ABE system that diminishes the reliance on the central authority. More specifically, in the scheme of [19], after the initial parameters are created, any party can be an authority and users can encrypt data based on any attributes issued by any authority. Zhang et al. [41] proposed a large universe multi-authority CP-ABE with white-box traceability in the prime order groups, which removes the limitation where traceable multi-authority CP-ABE cannot support large universe and achieves effective tracking of users who maliciously compromise keys.

All the aforementioned schemes are typical ABE schemes. Although they bear certain shortcomings, these ABE schemes open up a range of viable research directions and provide a foundation for subsequent research.

### 2.2. ABE Schemes with Outsourced Computation

With the continuous breakthroughs in cloud computing technology, leveraging cloud services to share the computational load for resource-constrained devices has emerged as a new research trend. It is essential to safeguard sensitive information when uploading data to cloud servers, since CSPs are not entirely trustworthy. Green et al. [42] first proposed an ABE scheme with outsourced decryption. More specifically, in the scheme of [42], for any ABE ciphertext satisfied by the set of user attributes, the cloud server converts these ciphertexts into constant size ElGamal ciphertexts using the transformation key without access to the contents of any ciphertext, and then sends them to the user. This scheme significantly reduces the computational cost on the user and ensures the confidentiality of the data. However, the scheme has a drawback: it cannot verify the integrity and correctness of the ElGamal ciphertexts. To address this issue, Lai et al. [43] refined the outsourced decryption verification function to improve the reliability of their scheme, at the cost of increased computation and communication. Lin et al. [44] constructed an ABE with verifiable outsourced decryption, based on attribute-based key encapsulation mechanism, symmetric-key encryption and commitment scheme decryption, which reduces the computation cost and the bandwidth by half. The outsourced decryption verification model of [44] can also be applied to CP-ABE. For example, Premkamal et al. [29] proposed a CP-ABE scheme that supports verifiable outsourced computing. This scheme limits the number of access requests a user can make, and it resists chosen-plaintext attacks, collusion attacks, and agent attacks, hence realizing big data privacy protection in cloud environments. Ge et al. [17] proposed an ABE scheme that supports reliable outsourced decryption. In this scheme, smart contracts are used to ensure that the decryption cloud server is rewarded when and only when it returns a correctly transformed ciphertext.

The above schemes reduce the computational cost on devices with the assistance of cloud computing. However, the total system computational cost is unchanged and remains very high since them all use bilinear pairing as the primary operation.

### 2.3. Pairing-Free ABE Schemes for IoT

In recent years, pairing-free ABE schemes have emerged as a significant research area to adapt lightweight ABE for resource-constrained devices in the IoT. Yao et al. [30] proposed an ECC-based ABE scheme and proved the security in an attribute-based choice set model under the ECDDH problem. This scheme aims to address the security and privacy of data in IoT, but has limitations in terms of access control granularity, extensibility and versatility. Odelu et al. [45] proposed an RSA-based CP-ABE scheme for cloud-based IoT applications. However, its use of an AND gate as the access structure makes it less expressive, restricting its application in scenarios requiring more complex access control policies. The pairing-free CP-ABE scheme for IoT proposed by Ding et al. [31] uses LSSS to achieve fine-grained access control. However, there are two challenges with this scheme. The first one is security, as the public key is not used for encryption, leading to the possibility that users who do not satisfy the access policy may be able to decrypt the ciphertext without the private key. The second one is feasibility, as the single fully trusted AA in the scheme needs to take on both the attribute management and outsourcing computing, which may result in overloading the AA with tasks, making the AA a bottleneck of the whole system.

Sowjanya et al. [46] proposed an ECC-based KP-ABE scheme, but it is not applicable to the IoT with a huge number of devices. Subsequently, Sowjanya and Dasgupta [32] designed an ECC-based CP-ABE scheme to handle private data in the IoT-based healthcare system. However, this scheme lacks scalability and versatility which makes it difficult to migrate to other scenarios. There are also some ECC-based ABE schemes (e.g., [34,35,47]) have been proposed, but the computational cost of them is expensive. Wang et al. [33] proposed a pairing-free CP-ABE scheme supporting attribute revocation for cloud-assisted smart grids, which, unlike the scheme of [31], can resist illegal key sharing attacks. However, this scheme only supports access tree, which reduces the flexibility of access control. In addition, the decryption cost and communication cost are significantly increased [34].

All of the above schemes construct lightweight ABE schemes by removing bilinear pairings, but they all have some shortcomings and thus need further improvement to make them truly effective pairing-free ABE schemes.

## 3. Preliminaries

### 3.1. Linear Secret Sharing Scheme

In ABE schemes, the data owner needs to set up access structures in order to control the access rights of other users. Available access structures include monotonic Boolean formulas, AND gate, access tree and LSSS. Each access structure has its own specific characteristics and advantages. Choosing an appropriate access structure can facilitate the targeted optimization and enhancement of the ABE scheme, in terms of computational speed, storage density, expression flexibility and control granularity. LSSS is one of the most commonly chosen access structures for recent CP-ABE schemes. Therefore, a brief description of LSSS is given here.

Denote the set of all integers from 0 to p−1 as $Z_{p}=\left\{0,1,\cdots ,p-1\right\}$. Let $P=\left\{P_{1},P_{2},\cdots ,P_{n}\right\}$ be a set of entities and Π be a secret sharing scheme over *P*. Π is an LSSS if it satisfies the following conditions.

(1) There exists a l×n shared matrix Λ for Π. Denote the *i*-th row vector of Λ as $\Lambda_{i}$. Each row of Λ corresponds to a mapping ρ(i) from the set $\left\{1,2,\cdots ,l\right\}$ onto *P*. Suppose that the secret is $s\in Z_{p}$. Randomly select $r_{2},r_{3},\cdots ,r_{n}\in Z_{p}$ and construct the column vector $v={\left(s,r_{2},r_{3},\cdots ,r_{n}\right)}^{T}$. Then $\Lambda_{i}v$ is the *l* secret component generated by *s* according to Π, and $\Lambda_{i}v$ corresponds to the entity ρ(i).
(2) All the components $\Lambda_{i}v$ form a one-dimensional vector on $Z_{p}$.

LSSS has linear reconfigurability. Suppose that Π is an LSSS for the access structure A. Given an authorized set S∈A and I={i:ρ(i)∈S}⊂{1,2,⋯,n}, then there exists a set of constants $c={\left\{c_{i}\in Z_{p}\right\}}_{i\in I}$ that can be found in polynomial time, such that $\sum_{i\in I}c_{i}\Lambda_{i}=\left(1,0,\cdots ,0\right)$. Furthermore, the secret *s* is recovered by $\sum_{i\in I}c_{i}\Lambda_{i}v=\left(1,0,\cdots ,0\right){\left(s,r_{2},r_{3},\cdots ,r_{n}\right)}^{T}=s$. If a set S∉A is given, then there does not exist a matching set of constants.

**Figure 1:**
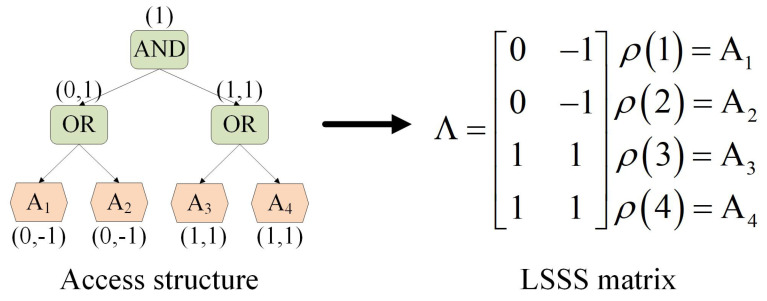
An example of the LSSS matrix representation access structure.

To make it easier for the reader to understand how matrix Λ enables flexible access control, an example is given here with reference to [30,31]. As shown in Figure 1, an access structure $A=\left(A_{1}\;\mathrm{OR}\;A_{2}\right)\mathrm{AND}\left(A_{3}\;\mathrm{OR}\;A_{4}\right)$ can be transformed into a 4 × 2 LSSS matrix Λ.

### 3.2. Formal Structure of CP-ABE

The standard CP-ABE consists of four algorithms, which are defined as follows.

(1) Setup(λ,U)→(PK,MSK): This algorithm is run by the AA, with the input security parameter λ and the full set of attributes *U*, to produce the output public parameter PK and the master secret key MSK.
(2) KeyGen(S,MSK)→SK: This algorithm is also operated by the AA and is responsible for generating the decryption key for all the users in the system. The algorithm inputs the set of user attributes *S* and the master secret key MSK, and outputs the secret key SK.
(3) $Enc\left((\Lambda ,\rho ),PK,m\right)\to CT$: The data owner encrypts plaintext by this algorithm. The algorithm inputs the access structure (Λ,ρ), the public parameter PK and the plaintext *m*, and outputs the ciphertext CT.
(4) Dec(CT,PK,SK)→mor⊥: The user decrypts the ciphertext by this algorithm. The algorithm inputs the ciphertext CT, the public parameters PK and the secret key SK. If the set of attributes *S* satisfies the access structure (Λ,ρ), the decryption succeeds and the plaintext *m* is outputted. Otherwise the decryption fails and the terminator ⊥ is outputted.

### 3.3. Elliptic Curve Decisional Diffie-Hellman Problem

**Definition** **1.** *Let G be a cyclic group and G is a generator of G. Given elements G, aG, bG and Z, where a,b∈G and Z may be equal to abG or a random value in G. The output is a judgment for Z=abG.*

The advantage of an algorithm B to solve the ECDDH problem can defined as

(1) $$\begin{array}{cc} \mathrm{Adv}(B)= & \frac{1}{2}\mathrm{Pr}\left[D\left(G,aG,bG,Z=abG\right)=1\right]\\ \; & +\frac{1}{2}\mathrm{Pr}\left[D\left(G,aG,bG,Z=R\right)=1\right]-\frac{1}{2}. \end{array}$$

## 4. System Architecture

### 4.1. System Model

**Figure 2:**
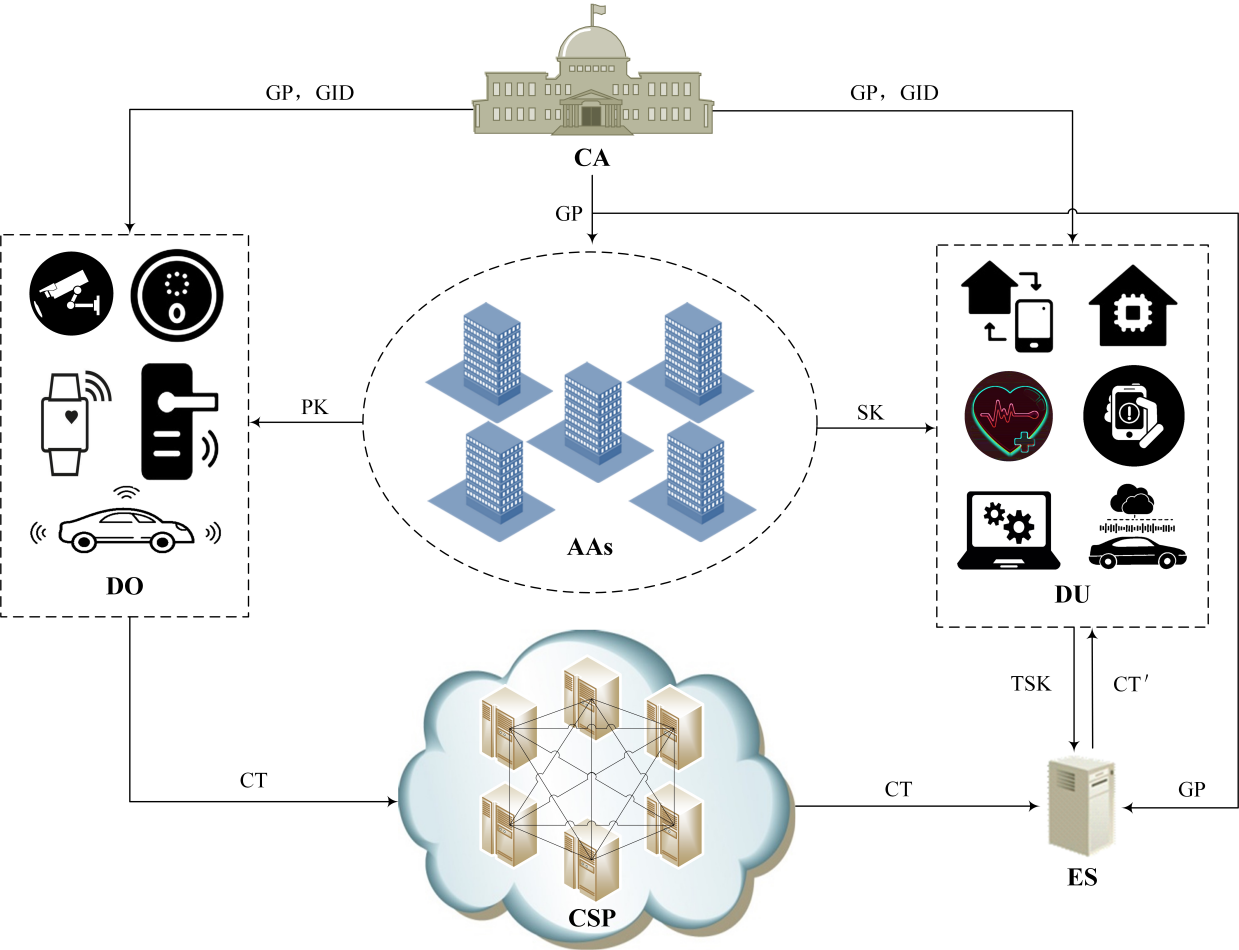
System model.

The proposed system model is depicted in Figure 2, which involves six entities, namely, central authority (CA), attribute authorities (AAs), data owner (DO), data user (DU), cloud service provider (CSP), and edge server (ES). The main functions of each entity are described below.

(1) CA: A fully trusted certification authority. It is unconditionally trusted by all the users, and is responsible for establishing the system and setting the global parameters. All the AAs and users must apply for registration with the CA. After successful registration, the CA will assign a globally unique identity to each of them. The CA will not be involved in the management of user attributes and distribution of keys during the process of data sharing.
(2) AAs: A set of fully trusted attribute authorities with the responsibility of distributing, updating and revoking attributes. In the system, IoT devices are users, and AAs set user attributes for them based on their identities and tasks. Our scheme adopts multi-authority to jointly manage user attributes, and none of the attributes managed by an AA overlaps with other AAs. During the initialization phase of the whole system and when a new device is connected to the system, each AA generates an attribute public–private key pair for the device based on the set of user attributes that it manages.
(3) DO: The owner of the data. DO creates an access policy that fits its security requirements based on the attribute fields defined in the system. The data that needs to be shared is then encrypted according to the access policy. Finally, the ciphertext is uploaded to the cloud and stored by the cloud server.
(4) DU: A requester of data. DU is a legitimate user in the system with an identity assigned by the CA and a set of attributes distributed by the AAs. DU can submit an access request to the CSP to download the ciphertext, and decrypt the ciphertext with the assistance of ES. Only the DUs that satisfy the access policy configured by the DO can successfully decrypt the ciphertext. However, DUs are not fully trustworthy. When the ciphertext cannot be decrypted independently, a DU may communicate privately with other users, team up with several users to assemble a set of attributes, and eventually launch a collusion attack in order to obtain the data.
(5) CSP: A third-party CSP that is not fully trusted. CSP has powerful computing resources and abundant storage space, and it provides some services to customers according to cooperation agreements and laws and regulations. In our scheme, CSP is the cloud side of the IoT, responsible for providing data storage services and access control services for IoT devices. DO can upload data that it cannot store and data that needs to be shared to CSP. DU has the right to apply for access to the CSP and download data after confirming their legal identity. Although CSP should protect the privacy of users while providing services, it may use data in order to analyze user portraits and mine valuable information. In addition, CSP may also be attacked by criminals, resulting in data leakage and tampering. Therefore, it is necessary to encrypt the data before uploading it to the CSP to ensure the security of private information.
(6) ES: ES does not have as rich storage space as CSP, but has higher computing power and greater energy supply than IoT devices, and is responsible for assisting DU in decrypting the ciphertext. DU sends a decryption request to ES and provides the transformation key, then ES downloads the relevant ciphertext from CSP and decrypts it. As ES can only perform most of the complex computation work in the decryption process, it cannot fully decrypt the ciphertext. Therefore, ES is unable to obtain the plaintext. The whole process transforms the ciphertext without revealing any data, and reduces the computational cost of IoT device.

### 4.2. The Overview of Proposed Scheme

**Figure 3:**
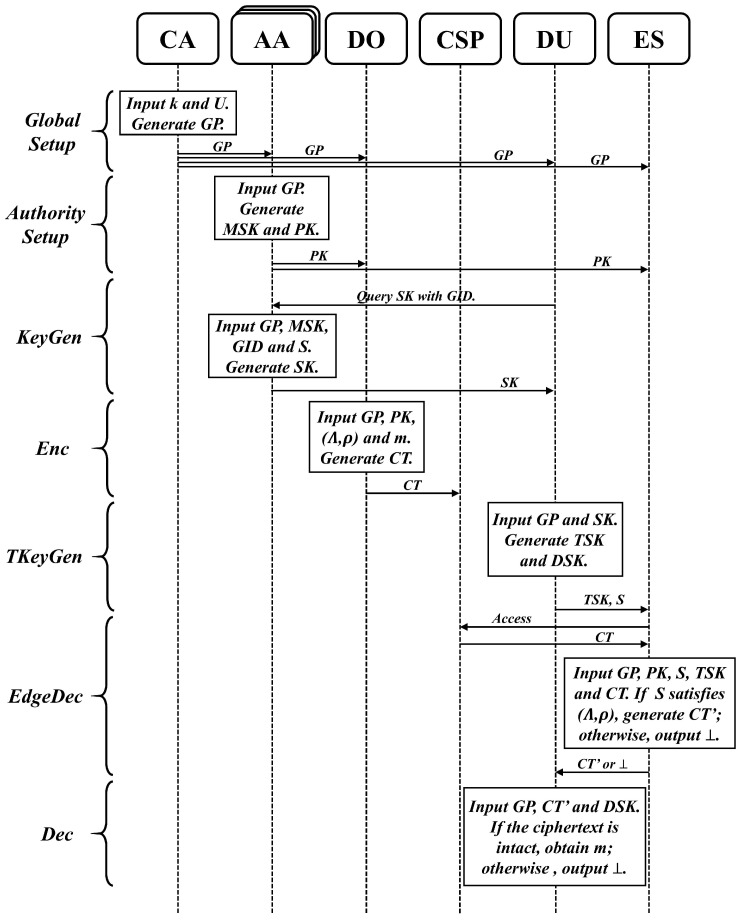
Overview of the proposed scheme.

An overview of the proposed scheme is depicted in Figure 3, which includes the seven algorithms of GlobalSetup, AuthoritySetup, KeyGen, KeyCon, Enc, EdgeDec, and Dec. A description of each algorithm is given below.

(1) GlobalSetup(k,U)→GP: The GlobalSetup algorithm is operated by CA, which inputs the system security parameters *k* and the full set of system attributes *U*, and then outputs the global parameters GP.
(2) AuthoritySetup(GP)→(PK,MSK): Assume that there are *n* AAs in the system $\left\{{\mathrm{AA}}_{1},{\mathrm{AA}}_{2},\cdots ,{\mathrm{AA}}_{n}\right\}$, and ${\mathrm{AA}}_{i}$ manages the set of attributes $U_{i}$. In this phase, AA runs the AuthoritySetup algorithm with the input system global parameters GP, to output the public key PK and the master private key MSK. The master private key MSK is stored by AA alone and the public key PK is published in the system.
(3) KeyGen(GP,MSK,GID,S)→SK: The KeyGen algorithm is operated by multiple AAs together. It inputs the global parameters GP, the master private key MSK, the user’s identifier GID and the set of attributes *S*, and then outputs the secret key SK.
(4) TKeyGen(GP,SK)→(TSK,DSK): DU runs the TKeyGen algorithm, inputting the global parameters GP and the secret key SK, and outputting the transformation key TSK and the decryption key DSK. ES can use TSK to perform an assisted decryption of the ciphertext, and then DU uses DSK to perform a final decryption.
(5) Enc(GP,PK,(Λ,ρ),m)→CT: The Enc algorithm is run by DO. It inputs the global parameters GP, the public key PK, the access structure (Λ,ρ) and the plaintext *m*, and then outputs the ciphertext CT.
(6) $EdgeDec\left(GP,PK,S,TSK,CT\right)\to CT^{\prime }\;\mathrm{or}\;⊥$: The EdgeDec algorithm is operated by ES with the inputs of the global parameters GP, the public key PK, the set of attributes *S*, the transformation key TSK and the ciphertext CT. If *S* satisfies (Λ,ρ), it outputs the transformed ciphertext $CT^{\prime }$. Otherwise, it outputs the termination symbol ⊥.
(7) $Dec(GP,CT^{\prime },DSK)\to m\;\mathrm{or}\;⊥$: The Dec algorithm is operated by DU with the inputs of the global parameters GP, the transformed ciphertext $CT^{\prime }$ and the decryption key DSK. If the integrity of the ciphertext is correct, the plaintext *m* is outputted. Otherwise the termination symbol ⊥ is outputted.

### 4.3. Security Model

The indistinguishability under chosen plaintext attack (IND-CPA) security of a CP-ABE scheme is commonly proved by an attack game between the adversary and the challenger [30,31,33]. The six phases of this game are presented as follows.

(1) Initialization: The adversary selects an access structure (Λ,ρ) to hand to the challenger.
(2) Setup: The challenger runs the GlobalSetup algorithm, generating the global parameters GP. Then the AuthoritySetup algorithm is run to generate the public key PK and the master private key MSK. Finally, GP and PK are sent to the adversary.
(3) Phase 1: The adversary selects a global identity GID and then acquires a legitimate set of attributes *S* from trusted AAs. However, it is required that none of the attributes in *S* satisfies the access structure (Λ,ρ). Then the adversary submits {GID,S} to the challenger and initiates a secret key query. After receiving {GID,S}, the challenger operates the KeyGen algorithm and returns the generated secret key SK to the adversary.
(4) Challenge: The adversary creates 2 equal length plaintexts $m_{0}$ and $m_{1}$ and submits $\left\{m_{0},m_{1}\right\}$ to the challenger. The challenger flips a random coin β∈{0,1} and runs the Enc algorithm to encrypt the plaintext $m_{\beta }$ according to the access structure (Λ,ρ). Finally it send the produced ciphertext CT to the adversary.
(5) The adversary repeats the steps in Phase 1.
(6) Guess: Based on the ciphertext CT, the adversary determines which of the plaintexts $m_{0}$ and $m_{1}$ the challenger has selected for encryption and gives a guess $\beta^{\prime }\in \left\{0,1\right\}$. The adversary wins the game when $\beta^{\prime }=\beta$.

In this game, $\mathrm{Pr}[\beta^{\prime }=\beta ]$ represents the probability of a correct guess, sand hence $\mathrm{Pr}\left[\beta^{\prime }=\beta \right]-\frac{1}{2}$ represents the opponent’s advantage in this game.

**Definition** **2.** *The scheme is chosen-plaintext attack secure if any polynomial-time adversary has at most a negligible advantage to win the above attack game.*

## 5. Proposed Scheme

We now provide the full technical details of our proposed pairing-free CP-ABE scheme. The notations and descriptions used in this section are summarized in Table 1.

GlobalSetup(k,U)→GP: After inputting the security parameter *k* and the full set of attributes *U* of the system, CA chooses a large prime *p*, and a finite field $GF\left(p\right)$ of order *p* according to *k*. Let *E* be an elliptic curve defined over the finite field $GF\left(p\right)$. Then, a point *G* of order *r* is chosen as a base point on *E*, and a cyclic group G on *E* is generated from *G*. Note that the elliptic curve discrete logarithm problem (ECDLP) on G is unsolvable in polynomial time. A strongly collision-resistant hash function $H:{\left\{0,1\right\}}^{*}\to Z_{r}^{*}$ is chosen, where $Z_{r}^{*}=Z_{r}\setminus \left\{0\right\}$, while ${\left\{0,1\right\}}^{*}$ indicates that the input message to *H* can be any length. Finally, CA generates the global parameter as Equation (2) and publishes it.

(2) $$GP=\left\{GF\left(p\right),E,G,U,H\right\}$$

AuthoritySetup(GP)→(PK,MSK): In the system, there are *n* AAs, each managing a particular set of attributes independently, and there is no overlap between any two sets of attributes. Each AA takes the input GP to run the AuthoritySetup algorithm. AA first chooses a pair of random values $x_{i},y_{i}\in Z_{r}$ for each attribute *i* according to the set of attributes that it manages. Then, AA generates the master private key as Equation (3) and the public key as Equation (4). Finally, AA keeps MSK and makes PK public in the system.

(3) $$MSK={\left\{x_{i},y_{i}\right\}}_{\forall i}$$

(4) $$PK={\left\{x_{i}G,y_{i}G\right\}}_{\forall i}$$

Additionally, AA maintains a list of attributes associated with the identifier GID for each legitimate user in the system.

KeyGen(GP,MSK,GID,S)→SK: This algorithm is run by all the AAs, and each AA generates the secret key for the user based on the set of attributes that it manages. The user submits GID to AA and requests a secret key, and AA takes the inputs GP, MSK and GID to run the KeyGen algorithm. AA first searches the list of attributes for the user based on GID. Then, AA generates the secret key as Equation (5) for each of the user’s attributes using the individually saved MSK. Finally, AA records the $SK_{GID,i}$ according to the user’s GID and attribute *i*. When the user applies for the secret key again, AA will directly return the recorded $SK_{GID,i}$.

(5) $$SK_{GID,i}={\left\{x_{i},H\left(GID\right)y_{i}\right\}}_{i\in S}$$

Enc(GP,PK,(Λ,ρ),m)→CT: Algorithm 1 shows the detailed flow of the Enc algorithm. The proposed scheme adopts a hybrid encryption approach, namely, using symmetric key encryption for the plaintext of the data to be shared, followed by asymmetric key encryption for the symmetric key. As a result, more than one data with the same access structure can be shared at a time. DU can request access to part or all of the data depending on the access requirements. DO defines the LSSS access structure (Λ,ρ), which specifies the attributes of DU who can access the data. Since DUs that can access data are no longer specified with identities, but are described using attributes, any DU that satisfies the LSSS access structure defined by the DO is a potential accessor. Therefore, the DO only needs to encrypt the data once before it can be shared to different DUs. It runs the Enc algorithm, inputting GP, PK, (Λ,ρ) and the plaintext $m=\{m_{1},m_{2},\cdots ,m_{f}\}$, where *m* includes *f* data files. DO picks a secret value sk∈G randomly and generates a symmetric key ck=H(sk). Then according to ck and the symmetric encryption algorithm $E_{ck}$, DO generates the ciphertext $\left\{M_{1},M_{2},\cdots ,M_{f}\right\}$ via Equation (6).
**Algorithm 1** Enc**Input:**  GP,PK,(Λ,ρ),m.**Output:**  CT. 1:$\{m_{1},m_{2},\cdots ,m_{f}\}=m$ 2:sk∈G 3:ck=H(sk) 4:**for** i∈[1,f] **do** 5:    $M_{i}=E_{ck}\left(m_{i},ck\right)$ 6:    $E_{i}=H\left(M_{i}\|ck\right)$ 7:**end for** 8:$s\in Z_{r}$ 9:$C_{0}=sk+sG$10:$u=\left(s,u_{2},u_{3},\cdots ,u_{n}\right)\in Z_{r}^{n}$, $v=\left(0,v_{2},v_{3},\cdots ,v_{n}\right)\in Z_{r}^{n}$11:$\lambda_{i}=\Lambda_{i}u$, $\omega_{i}=\Lambda_{i}v$12:**for** i∈[1,l] **do**13:    $\alpha_{i},\beta_{i},\gamma_{i}\in Z_{r}$14:    $C_{1,i}=\lambda_{i}-\alpha_{i}\gamma_{i}$, $C_{2,i}=\omega_{i}-\beta_{i}\gamma_{i}$, $C_{3,i}=\gamma_{i}G$, $C_{4,i}=\left(x_{i}+\alpha_{i}\right)C_{3,i}$, $C_{5,i}=\left(y_{i}+\beta_{i}\right)C_{3,i}$15:**end for**16:$CT=\left\{\left(\Lambda ,\rho \right),{\left\{M_{i},E_{i}\right\}}_{i\in \{1,\cdots ,f\}},C_{0},{\left\{C_{1,j},C_{2,j},C_{3,j},C_{4,j},C_{5,j}\right\}}_{j\in \{1,\cdots ,l\}}\right\}$17:**return** CT

(6) $$M_{i}=E_{ck}\left(m_{i},ck\right),i\in \left[1,f\right]$$

In order for DU to verify the integrity of the data ciphertext and prevent the data ciphertext from being tampered and replaced, the verification data needs to be generated. For $\left\{M_{1},M_{2},\cdots ,M_{f}\right\}$, DO computes the verification data $\left\{E_{1},E_{2},\cdots ,E_{f}\right\}$ via Equation (7).

(7) $$E_{i}=H\left(M_{i}\|ck\right),i\in \left[1,f\right]$$

To blind the secret value sk, DO select a random value $s\in Z_{r}$ and computes a component of the ciphertext $C_{0}=sk+sG$. Let $Z_{r}^{n}$ represent the set of *n*-dimensional vectors with all the elements of each vector belonging to $Z_{r}$. DO randomly chooses two vectors as Equation (8), whose first elements are *s* and 0, respectively.

(8) $$\begin{array}{c} u=\left(s,u_{2},u_{3},\cdots ,u_{n}\right)\in Z_{r}^{n}\\ v=\left(0,v_{2},v_{3},\cdots ,v_{n}\right)\in Z_{r}^{n} \end{array}$$

Next, DO computes $\lambda_{i}=\Lambda_{i}u$ and $\omega_{i}=\Lambda_{i}v$, where $\forall i\in \left\{1,2,\cdots ,l\right\}$ and $\Lambda_{i}$ is the *i*th row of the access control matrix. Afterward, DO randomly selects $\alpha_{i},\beta_{i},\gamma_{i}\in Z_{r}$ and computes the rest component of the ciphertext via Equation (9). Eventually, DO generates a complete ciphertext as Equation (10) and send it to CSP for storage.

(9) $$\begin{array}{c} C_{1,i}=\lambda_{i}-\alpha_{i}\gamma_{i}\\ C_{2,i}=\omega_{i}-\beta_{i}\gamma_{i}\\ C_{3,i}=\gamma_{i}G\\ C_{4,i}=\left(x_{i}+\alpha_{i}\right)C_{3,i}\\ C_{5,i}=\left(y_{i}+\beta_{i}\right)C_{3,i} \end{array}$$

(10) $$CT=\left\{\left(\Lambda ,\rho \right),{\left\{M_{i},E_{i}\right\}}_{i\in \{1,\cdots ,f\}},C_{0},{\left\{C_{1,j},C_{2,j},C_{3,j},C_{4,j},C_{5,j}\right\}}_{j\in \{1,\cdots ,l\}}\right\}$$

TKeyGen(GP,SK)→(TSK,DSK): DU runs the TKeyGen algorithm after obtaining the SK from AA. AA chooses the random number $z\in Z_{r}$ as the decryption key and generates the transformation key $TSK={\left\{TSK_{i}\right\}}_{i\in S}$ as Equtaion (11). DU saves the decryption key $DSK=\left\{z\right\}$ alone for the final decryption phase to obtain the plaintext, sends the transformation key TSK to ES and entrusts ES with the assisted decryption of the ciphertext.

(11) $$TSK_{i}=x_{i}+H\left(GID\right)y_{i}z$$

$EdgeDec\left(GP,PK,S,TSK,CT\right)\to CT^{\prime }\;\mathrm{or}\;⊥$: After receiving TSK and *S* from DU, ES downloads the specified ciphertext CT from CSP.

If *S* satisfies (Λ,ρ), then a set of constants $c=\left\{c_{1},c_{2},\cdots ,c_{l}\right\}\in Z_{r}^{l}$ can be found in polynomial time, which satisfies $\sum_{i\in S}c_{i}\Lambda_{i}=\left(1,0,0,\cdots ,0\right)$. Therefore, ES can perform Equation (12) to partially decrypt ciphertext and generate the transformed ciphertext as Equation (13). Finally, the transformed ciphertext $CT^{\prime }$ is sent to DU.

(12) $$\begin{array}{c} C_{1,i}^{\prime }=C_{1,i}G+C_{4,i}\\ C_{2,i}^{\prime }=H\left(GID\right)\left(C_{2,i}G+C_{5,i}\right)\\ C_{3,i}^{\prime }=C_{3,i}TSK_{i}\\ CT_{1}=\sum_{i\in S}c_{i}C_{1,i}^{\prime }\\ CT_{2}=\sum_{i\in S}c_{i}C_{2,i}^{\prime }\\ CT_{3}=\sum_{i\in S}c_{i}C_{3,i}^{\prime } \end{array}$$

(13) $${\mathrm{CT}}^{\prime }=\left\{{\left\{M_{i},E_{i}\right\}}_{i\in \{1,\cdots ,f\}},C_{0},CT_{1},CT_{2},CT_{3}\right\}$$

If *S* does not satisfy (Λ,ρ), the algorithm will terminate and send ⊥ to DU.

$Dec(GP,CT^{\prime },DSK)\to m$: DU first computes the secret value via Equation (14) after receiving ${\mathrm{CT}}^{\prime }$. Then, DU generates the symmetric key ck=H(sk). Next, the integrity of $\left\{M_{1},M_{2},\cdots ,M_{f}\right\}$ is verified against $\left\{E_{1},E_{2},\cdots ,E_{f}\right\}$ to determine whether the data has been tampered with and replaced. Specifically, ∀i∈{1,2,⋯,f}, DU computes $E_{i}^{\prime }=H\left(M_{i}\|ck\right)$ and compares it with $E_{i}$. If $E_{i}=E_{i}^{\prime }$, which proves that $M_{i}$ is complete, it decrypts $M_{i}$ by ck and recovers the plaintext $m_{i}$. If $E_{i}\neq E_{i}^{\prime }$, $M_{i}$ is proved to be incomplete and ⊥ is returned. There may be a number of incomplete data, for which DU only needs to make another access request, without having to repeatedly access the data it has obtained.

(14) $$sk=C_{0}-\left(CT_{1}+zCT_{2}-CT_{3}\right)$$

## 6. Security Analysis

### 6.1. Correctness Proof

In the proposed scheme, we define data plaintext $\left\{m_{1},m_{2},\cdots ,m_{f}\right\}$, data ciphertext $\left\{M_{1},M_{2},\cdots ,M_{f}\right\}$, verification data $\left\{E_{1},E_{2},\cdots ,E_{f}\right\}$, access structure (Λ,ρ), ciphertext CT, attribute set *S* of DU, transformation key TSK and decryption key DSK. The correctness of the proposed scheme can be proved as follows.

First we verify the EdgeDec algorithm. Based on the ciphertext CT, ES can compute:

(15) $$\begin{array}{cc} C_{1,i}^{\prime } & =C_{1,i}G+C_{4,i}=\left(\lambda_{i}-\alpha_{i}\gamma_{i}\right)G+\left(x_{i}+\alpha_{i}\right)C_{3,i}\\ & =\left(\lambda_{i}-\alpha_{i}\gamma_{i}\right)G+\left(x_{i}+\alpha_{i}\right)\gamma_{i}G=\left(\lambda_{i}+x_{i}\gamma_{i}\right)G, \end{array}$$

(16) $$\begin{array}{cc} C_{2,i}^{\prime }= & H(GID)\left(C_{2,i}G+C_{5,i}\right)\\ = & H(GID)\left(\left(\omega_{i}-\beta_{i}\gamma_{i}\right)G+\left(y_{i}+\beta_{i}\right)C_{3,i}\right)\\ = & H(GID)\left(\left(\omega_{i}-\beta_{i}\gamma_{i}\right)G+\left(y_{i}+\beta_{i}\right)\gamma_{i}G\right)\\ = & H(GID)\left(\omega_{i}+y_{i}\gamma_{i}\right)G, \end{array}$$

(17) $$\begin{array}{cc} C_{3,i}^{\prime }= & C_{3,i}TSK_{i}=\left(x_{i}+H\left(GID\right)y_{i}z\right)\gamma_{i}G. \end{array}$$

If *S* satisfies (Λ,ρ), then $\exists c=\left(c_{1},c_{2},\cdots ,c_{l}\right)\in Z_{r}^{l}$ such that $\sum_{i\in S}c_{i}\Lambda_{i}=\left(1,0,0,\cdots ,0\right)$ Then:

(18) $$\begin{array}{cc} \sum_{i\in S}c_{i}\lambda_{i} & =\sum_{i\in S}c_{i}\Lambda_{i}u\\ & =\left(1,0,0,\cdots ,0\right)\cdot {\left(s,u_{2},u_{3},\cdots ,u_{n}\right)}^{T}=s, \end{array}$$

(19) $$\begin{array}{cc} \sum_{i\in S}c_{i}\omega_{i} & =\sum_{i\in S}c_{i}\Lambda_{i}vs.\\ & =\left(1,0,0,\cdots ,0\right)\cdot {\left(0,v_{2},v_{3},\cdots ,v_{n}\right)}^{T}=0. \end{array}$$

ES next computes:

(20) $$\begin{array}{cc} CT_{1} & =\sum_{i\in S}c_{i}C_{1,i}^{\prime }=\sum_{i\in S}c_{i}\left(\lambda_{i}+x_{i}\gamma_{i}\right)G\\ & =\sum_{i\in S}c_{i}\lambda_{i}G+\sum_{i\in S}c_{i}x_{i}\gamma_{i}G=sG+\sum_{i\in S}c_{i}x_{i}\gamma_{i}G, \end{array}$$

(21) $$\begin{array}{cc} CT_{2} & =\sum_{i\in S}c_{i}C_{2,i}^{\prime }=\sum_{i\in S}c_{i}H\left(GID\right)\left(\omega_{i}+y_{i}\gamma_{i}\right)G\\ & =H\left(GID\right)\left(\sum_{i\in S}c_{i}\omega_{i}G+\sum_{i\in S}c_{i}y_{i}\gamma_{i}G\right)\\ & =H\left(GID\right)\sum_{i\in S}c_{i}y_{i}\gamma_{i}G, \end{array}$$

(22) $$\begin{array}{cc} CT_{3} & =\sum_{i\in S}c_{i}C_{3,i}^{\prime }=\sum_{i\in S}c_{i}\left(x_{i}+H\left(GID\right)y_{i}z\right)\gamma_{i}G\\ & =\sum_{i\in S}c_{i}x_{i}\gamma_{i}G+H\left(GID\right)\sum_{i\in S}c_{i}y_{i}\gamma_{i}zG. \end{array}$$

Next, we verify the Dec algorithm. According to $CT^{\prime }$, DU can compute:

(23) $$\begin{array}{cc} \; & C_{0}-\left(CT_{1}+zCT_{2}-CT_{3}\right)=\left(sk+sG\right)\\ \; & \;-(\left(sG+\sum_{i\in S}c_{i}x_{i}\gamma_{i}G\right)+zH\left(GID\right)\sum_{i\in S}c_{i}y_{i}\gamma_{i}G\\ \; & \;-\left(\sum_{i\in S}c_{i}x_{i}\gamma_{i}G+H\left(GID\right)\sum_{i\in S}c_{i}y_{i}\gamma_{i}zG\right))\\ \; & \;=sk+sG-sG=sk. \end{array}$$

ck can be mapped by the elliptic curve *E* and the point sk. DU computes $E_{i}^{\prime }=H\left(M_{i}\|ck\right)$, ∀i∈{1,2,⋯,f}. If the data ciphertext is integral, $E_{i}^{\prime }=E_{i}$. Finally, $M_{i}$ is decrypted using ck to get $m_{i}$. By now, DU successfully accesses to the information.

### 6.2. Security Proof

We next prove that the proposed scheme achieves the CPA security under the ECDDH problem.

**Theorem** **1.** *Since the ECDDH problem is hard to solve, no polynomial-time adversary can break our scheme.*

**Proof.**  First, if there exists an adversary A that can break our scheme in polynomial time with a non-negligible advantage ε>0, then there exists an effective algorithm B that can distinguish an ECDDH tuple from a random tuple in polynomial time with the advantage $\frac{\varepsilon }{2}>0$. In the attack game, algorithm B will be constructed as a simulator D.

Challenger C selects an elliptic curve *E* defined over a finite field GF(p), where the order of GF(p) is *p*. The point *G* on *E* is chosen as the base point and the order of *G* is *r*. A cyclic group G on *E* is generated from *G*, and the elliptic curve discrete logarithm problem (ECDLP) in G is impossible to solve in polynomial time. Then, challenger C randomly chooses β∈{0,1}, $a,b\in Z_{r}$ and R∈G. If β=0, set (G,aG,bG,abG) to the tuple (G,aG,bG,Z); Otherwise, set (G,aG,bG,R) to the tuple (G,aG,bG,Z). Then the tuple (G,aG,bG,Z) is delivered to simulator D, and simulator D interacts with adversary A in the following game.

Initialization: Adversary A sets up an access structure (Λ,ρ) and sends it to simulator D. (Λ,ρ) is to be challenged.

Setup: Simulator D first runs the GlobalSetup(k,U) algorithm in the original scheme with a security parameter *k* and a full set of attributes *U* provided by challenger C to generate the global parameter $GP=\left\{GF\left(p\right),E,G,U,H\right\}$. Then, simulator D selects $x_{i},y_{i}\in Z_{r}$ for each attribute i∈U, and set the public key $PK={\left\{x_{i}aG,y_{i}aG\right\}}_{i\in U}$. Finally, GP and PK are given to adversary A, and the master private key $MSK={\left\{x_{i},y_{i}\right\}}_{i\in U}$ is kept by simulator D alone.

Phase 1: Adversary A randomly chooses a GID, and then applies valid attributes to any number of all trusted AAs to make up its own attribute set *S*. However, it requires that adversary A should avoid the attributes used in (Λ,ρ) when choosing attributes, in order to restrict that there are no attributes in *S* that can satisfy (Λ,ρ). Simulator D then starts accepting queries for the secret key initiated by adversary A. Simulator D responds according to the attributes corresponding to adversary A’s GID in the attribute list and returns the generated secret key SK to adversary A.

Challenge: Adversary A generates two plaintexts of equal length, $m_{0}$ and $m_{1}$, and sends them to D. Simulator D chooses two random vectors $u=\left(s,u_{2},u_{3},\cdots ,u_{n}\right)\in Z_{r}^{n}$ and $v=\left(0,v_{2},v_{3},\cdots ,v_{n}\right)\in Z_{r}^{n}$ whose first elements are the cryptographic index *s* and 0, respectively. ∀i∈{1,2,⋯,l}, it computes $\lambda_{i}=\Lambda_{i}u$ and $\omega_{i}=\Lambda_{i}v$, where $\Lambda_{i}$ is the *i*th row of the access control matrix. Then D randomly selects β∈{0,1} and encrypts the plaintext $m_{\beta }$ according to (Λ,ρ) to generate the ciphertext CT. Specifically, D computes $C_{0}$ with the $Enc(GP,PK,\left(\Lambda ,\rho \right),m_{\beta })$ in the original scheme. Subsequently, D selects $\alpha_{i},\beta_{i},\gamma_{i}\in Z_{r}$ and sets $C_{1,i}=\lambda_{i}-\alpha_{i}\gamma_{i}$, $C_{2,i}=\omega_{i}-\beta_{i}\gamma_{i}$, $C_{3,i}=\gamma_{i}Z$, $C_{4,i}=\gamma_{i}x_{i}aG+\alpha_{i}\gamma_{i}bG$ and $C_{5,i}=\gamma_{i}y_{i}aG+\beta_{i}\gamma_{i}bG$. Finally, CT is sent to adversary A.

Phase 2: Repeat the operation of Phase 1 under the same constraints.

Guess: Adversary A chooses $\beta^{\prime }\in \left\{0,1\right\}$ as a guess for β.

If $\beta^{\prime }=\beta$, simulator D outputs 1 to represent the guess result of Z=abG. In this case, the the adversary A successfully guesses the plaintext $m_{\beta }$, and thus wins the attack game. Otherwise, D outputs 0 to represent the guess result of Z=R.

Here, the probability of adversary A winning is defined as $\mathrm{Pr}[\beta^{\prime }=\beta ]$, and its advantage is given by $\mathrm{Pr}\left[\beta^{\prime }=\beta \right]-\frac{1}{2}$.

When Z=abG, since adversary A has the advantage of ε>0, the probability that simulator D guesses correctly for β is

(24) $$\mathrm{Pr}\left[D\left(G,aG,bG,Z=abG\right)=1\right]=\frac{1}{2}+\varepsilon .$$

When Z=R, since *R* is chosen randomly, adversary A’s guess for β does not have any advantage. Simulator D’s guess for β fits the Bernoulli distribution at this time, and the probability of a correct guess is

(25) $$\mathrm{Pr}\left[D\left(G,aG,bG,Z=R\right)=1\right]=\frac{1}{2}.$$

Therefore, the probability that simulator D succeeds is

(26) $$\begin{array}{cc} \mathrm{Adv}(D)= & \frac{1}{2}\mathrm{Pr}\left[D\left(G,aG,bG,Z=abG\right)=1\right]\\ \; & +\frac{1}{2}\mathrm{Pr}\left[D\left(G,aG,bG,Z=R\right)=1\right]-\frac{1}{2}\\ = & \frac{1}{2}\left(\frac{1}{2}+\varepsilon \right)+\frac{1}{2}\times \frac{1}{2}-\frac{1}{2}=\frac{\varepsilon }{2}. \end{array}$$

Thus, simulator D distinguishes an ECDDH tuple from a random tuple by the advantage of ε/2. Because ε is non-negligible, the advantage $\frac{\varepsilon }{2}$ of D is also non-negligible.

However, there exists no effective algorithm that can solve the ECDDH problem, and thus adversary A does not exist. Therefore, the proposed scheme has the indistinguishability under chosen-plaintext attack (IDN-CPA). □

### 6.3. Resistant to Collusion Attack

In order to guarantee effective access control to the data, the scheme must be able to resist collusion attacks. In other words, more than one user cannot decrypt the ciphertext independently because their attributes does not satisfy the access structure (Λ,ρ). When these users collude with each other to piece together the attribute sets, they still cannot successfully decrypt the ciphertext. In the proposed scheme, CA binds a GID for each legitimate user in the system, and AA associates the attribute list with GID, so that different users cannot piece together attributes with each other for decryption because of different GID in the decryption process.

For example, consider two legitimate users, Alice and Bob, in the system, and a ciphertext associated with access structure ${attr}_{\varphi }\wedge {attr}_{\psi }$. Alice has the attribute ${attr}_{\varphi }$ and Bob has the attribute ${attr}_{\psi }$. Obviously, both Alice and Bob do not satisfy the access structure and cannot decrypt the ciphertext independently. Therefore, Alice and Bob collude together and try to access the ciphertext by sharing attributes. During the decryption process, $\forall i\in \left\{{attr}_{\varphi },{attr}_{\psi }\right\}$, Alice will get the final ciphertext computed by ES as follows:

(27) $$\left\{\begin{array}{c} C_{1,i,Alice}^{\prime }\;=\;\lambda_{i}G\;+\;x_{i}\gamma_{i}G,\\ C_{2,i,Alice}^{\prime }\;=\;H\left(GID_{Alice}\right)\omega_{i}G\;+\;H\left(GID_{Alice}\right)y_{i}\gamma_{i}G,\\ C_{3,i,Alice}^{\prime }\;=\;x_{i}\gamma_{i}G\;+\;H\left(GID_{Alice}\right)y_{i}\gamma_{i}zG. \end{array}\right.$$

Bob will get the final ciphertext computed by ES as follows:

(28) $$\left\{\begin{array}{c} C_{1,i,Bob}^{\prime }\;=\;\lambda_{i}G\;+\;x_{i}\gamma_{i}G,\\ C_{2,i,Bob}^{\prime }\;=\;H\left(GID_{Bob}\right)\omega_{i}G\;+\;H\left(GID_{Bob}\right)y_{i}\gamma_{i}G,\\ C_{3,i,Bob}^{\prime }\;=x_{i}\gamma_{i}G\;+\;H\left(GID_{Bob}\right)y_{i}\gamma_{i}zG. \end{array}\right.$$

If a user has attributes that satisfy the access structure ${attr}_{\varphi }\wedge {attr}_{\psi }$, a set of constants *c* can be computed in polynomial time to enable linear reconstruction. However, because Alice and Bob have different identifiers GID, it results in $H\left({GID}_{Alice}\right)\neq H\left({GID}_{Bob}\right)$. This difference prevents them from colluding to compute sG, thereby ensuring they cannot decrypt the ciphertext together. Therefore, our scheme can effectively resists collusion attacks.

### 6.4. Data Confidentiality

AA chooses a pair of random values $x_{i},y_{i}\in Z_{r}$ for each attribute *i* as the private key for attribute *i*. Then it generates the master private key $MSK={\left\{x_{i},y_{i}\right\}}_{\forall i}$ and the public key $PK={\left\{x_{i}G,y_{i}G\right\}}_{\forall i}$. MSK is kept by AA alone. Since there does not exist an efficient algorithm that can break ECDLP in polynomial time, any user who has PK cannot obtain additional information about MSK. After encrypting the data plaintext *m* into data ciphertext *M* by symmetric key ck, DO maps ck to a point sk on the elliptic curve *E* and hides sk in $C_{0}=sk+sG$. Since the blinding index $s\in Z_{r}$ is chosen randomly and kept by DO alone, it is hard for other users to conjecture the blinding factor sG. So other users are unable to separate sG from $C_{0}$, and cannot get sk, let alone decrypt *M*. DO construct *s* in the random vector $u=\left(s,u_{2},u_{3},\cdots ,u_{n}\right)\in Z_{r}^{n}$. Then, according to the set access structure (Λ,ρ), *u* is divided into $\lambda_{i}=\Lambda_{i}u$, i∈{1,2,⋯,l} by LSSS. In the decryption process, DU that can find an appropriate set of constants $c=\left\{c_{1},c_{2},\cdots ,c_{l}\right\}\in Z_{r}^{l}$ in polynomial time to satisfy $\sum_{i\in S}c_{i}\Lambda_{i}=\left(1,0,0,\cdots ,0\right)$, making $\sum_{i\in S}c_{i}\lambda_{i}=s$, must have a set of attributes that meet (Λ,ρ). However, other DUs, who do not have the attributes meeting (Λ,ρ), cannot recover *s* in polynomial time, and consequently cannot continue to decrypt.

## 7. Performance Analysis

### 7.1. Comparison of Features

In order to analyze the achievable performance of our proposed scheme, we first compare its features with those of the existing schemes [19,30,31,33,41] in Table 2. Features include three categories: infrastructure, lightweightness and security. The infrastructure includes access structures and multiple authorities. Lightweightness compares whether the scheme uses a pairing-free design, supports outsourced decryption and the verifiability of outsourced decryption. Security compares resistance to collusion attacks and provable security.

As shown in Table 2, the schemes [19,31,41] support LSSS access structure and have more fine-grained access control than the schemes with access tree [30,33]. The schemes [19,41] implement multi-authority, but both use expensive bilinear pairing operations. Additionally, the scheme [19] is under the static assumption, while the scheme [41] is under the assumption of the q-decisional parallel bilinear Diffie–Hellman exponent 2 problem (q-DPBDHE2). The schemes [30,31,33] are pairing-free lightweight schemes that use elliptic curve scalar multiplication as the base operation. Moreover, they are IND-CPA under the ECDDH assumption. The scheme [31] supports LSSS access structure and outsourced decryption, which can effectively reduce the computational cost of DU while providing fine-grained access control. However, this scheme does not support verifiable outsourced decryption, lacks multi-authority support, and faces the risk of illegal key sharing. The scheme [33] supports an access tree structure and resists collusion attacks, but it does not include multi-authority support or outsourced decryption capabilities. Among all the schemes compared in Table 2, only our scheme possesses all the desired features, namely, avoiding bilinear pairing, supporting multi-authority, verifiable outsourced decryption and LSSS access structure, resisting collusion attacks, and being IDN-CPA under the ECDDH assumption.

### 7.2. Comparison of Computation Costs

We next compare the computation costs of our scheme with those of the existing schemes [19,30,31,33,41]. Computational complexity of a scheme is mainly concerned with the major operations performed during encryption and decryption. The basic operations include bilinear pairing operation, denoted as P, which is the most expensive operation, two types of modulo power operation, denoted as E and $E_{T}$, and scalar multiplication operation, denoted as S. Compared with these three operations, other lightweight operations can be ignored, such as arithmetic operations, point-addition operations, hashing operations, encryption and decryption of symmetric key encryption.

Table 3 compares the computation costs for the six schemes, where *n* is the number of all attributes in the system, *l* is the number of attributes in the access structure, and *u* is the number of attributes of the user. According to [30,32], scalar multiplication (S) can be used as the unit for comparing the computation costs. Since the bilinear pairing operations in the schemes [19,41] are symmetric, one bilinear pairing operation is approximately equal to three scalar multiplication operations. A modulo power operation is approximately equal to a scalar multiplication operation. According to the setting of the number of attributes in these comparing schemes, we assume n=30, l=10 and u=5. Note that the encryption and decryption costs in the table are local costs and do not include computing costs outsourced to edge servers and the cloud.

As shown in Table 3, the computation costs of the schemes based on bilinear pairing [19,41] are much higher than the pairing-free schemes. Among the pairing-free schemes, the encryption cost of our scheme is slightly reduced compared with the schemes [31,33], but is higher than that of the scheme [30]. For decryption, the number of scalar multiplication operations required in the schemes [30,31,33] is linearly related to the number of attributes, while our scheme only needs single scalar multiplication operation, which is the minimum and independent of the number of attributes.

In the IoT environment, as more devices are added, the attributes describing them must be refined to accommodate their diversity and heterogeneity. This will increase the number of attributes in the system, which, in turn, increases the decryption complexity of the schemes [19,30,31,33,41]. By contrast, the decryption complexity of our scheme remains to be the minimum of 1S. Moreover, in the process of sharing one copy of data, the encryption algorithm needs to be performed only once by the data owner, while all the legitimate devices in the IoT are potential users, and any device that needs to access the data will need to decrypt the ciphertext. Consequently, the decryption algorithm will be performed many times. Therefore, the optimization of the decryption algorithm is more beneficial to reduce the overall computational burden of the system. Compared to other schemes [19,30,31,33,41], our scheme is much more efficient and better suited for the IoT environment.

## 8. Conclusions

In this paper, we proposed a secure and lightweight CP-ABE scheme without pairing, designed to facilitate controllable one-to-many data sharing among IoT devices. The proposed scheme sets multiple attribute authorities to decentralize attribute management, reducing the burden on a single attribute authority and avoiding the bottlenecks associated with single-point systems. To minimize computational complexity, the proposed scheme adopts a pairing-free construction, thereby avoiding the intensive computations required by bilinear pairing operations. Furthermore, by offloading most of the complex decryption tasks to an edge server, the decryption cost for data users is minimized, i.e., only one scalar multiplication operation needs to be performed. Thus, the proposed scheme is particularly suitable for IoT environments with limited computational resources. Security analysis has proved that the proposed scheme is IDN-CPA under the ECDDH assumption and resistant to collusion attacks. The performance analysis concluded that our scheme not only provides more features, but is also more computationally efficient than existing alternatives. In particular, while current unpaired attribute-based encryption schemes have linear decryption costs, our scheme achieves a constant decryption cost. In future work, we plan to extend this scheme to ensure the non-repudiation of cloud, edge, and user interactions through the integration of blockchain technology.

## Figures and Tables

**Table 1:** Abbreviations and notations.

Notation	Descriptions
*k*	Security parameter
*U*	attribute set of system
G	cyclic group
*G*	Generator of G
*H*	Hash function
GP	Global parameter
GID	Identifier
MSK	Master private key
$x_{i},y_{i}$	Master private key components for attribute *i*
PK	Public key
$x_{i}G,y_{i}G$	Public key components for attribute *i*
$SK_{GID,i}$	Secret key of user GID for attribute *i*
sk	Secret value
ck	Symmetric key
$E_{ck}$	symmetric encryption algorithm with ck
*m*	Plaintext containing *f* data files
$m_{i}$	*i*th data file
$M_{i}$	Symmetric encrypted ciphertext of $m_{i}$
$E_{i}$	Verification data of $M_{i}$
u,v	*n*-dimensional vectors
CT	ABE ciphertext
$C_{0},C_{1,i},C_{2,i},C_{3,i},C_{4,i},C_{5,i}$	Components of the ciphertext CT
$TSK_{i}$	Transformation key for attribute *i*
DSK	Decryption key
$CT^{\prime }$	Transformation ciphertext

**Table 2:** Comparison of features for various schemes.

Scheme	Infrastructure		Lightweightness			Security	
	Access Structure	Multi-Authority	Pairing Free	Outsourced Decryption	Verifiable Outsourcing	Resistant to Collusion Attack	Provable Security
Lewko [19]	LSSS	✓	✗	✗	–	✓	fully security
Zhang [41]	LSSS	✓	✗	✗	–	✗	IND-CPA
Yao [30]	Access tree	✗	✓	✗	–	✗	IND-CPA
Ding [31]	LSSS	✗	✓	✓	✗	✓	IND-CPA
Wang [33]	Access tree	✗	✓	✗	–	✓	IND-CPA
Our Scheme	LSSS	✓	✓	✓	✓	✓	IND-CPA

**Table 3:** Comparison of computation costs for various schemes.

Scheme	Encryption	Decryption
Lewko [19]	(5l+1)E≈51S	nE+2nP≈210S
Zhang [41]	$6l\;E+\left(2l+1\right)\;E_{T}$ +(2l+1)P≈144S	3lE+3lP≈120S
Yao [30]	(l+1)S≈11S	(u+1)S≈6S
Ding [31]	(4l+1)S≈41S	(u+1)S≈6S
Wang [33]	(3l+2)S≈32S	(3l+1)S≈31S
Our Scheme	(3l+1)S≈31S	1S

## Data Availability

Data are contained within the article.
